# Toward a science of human–AI teaming for decision making: A complementarity framework

**DOI:** 10.1093/pnasnexus/pgag030

**Published:** 2026-02-19

**Authors:** Cleotilde Gonzalez, Kate Donahue, Daniel G Goldstein, Hoda Heidari, Mohammad S Jalali, Beau Schelble, Aarti Singh, Anita Williams Woolley

**Affiliations:** Social and Decision Sciences Department, Carnegie Mellon University, 5000 Forbes Avenue, Pittsburgh, PA 15213, USA; Software and Societal Systems Department, Carnegie Mellon University, 5000 Forbes Avenue, Pittsburgh, PA 15213, USA; Computer Science Department, Massachusetts Institute of Technology, University of Illinois at Urbana-Champaign, 601 E. John Street, Champaign, IL 61820, USA; Microsoft Research, 300 Lafayette Street, New York, NY 10012, USA; Machine Learning Department, Carnegie Mellon University, 5000 Forbes Avenue, Pittsburgh, PA 15213, USA; Software and Societal Systems Department, Carnegie Mellon University, 5000 Forbes Avenue, Pittsburgh, PA 15213, USA; Harvard Medical School, Harvard University, 25 Shattuck Street, Boston, MA 02115, USA; Industrial and Systems Engineering, University of Tennessee at Knoxville, 527 Andy Holt Tower, Knoxville, TN 37996, USA; Machine Learning Department, Carnegie Mellon University, 5000 Forbes Avenue, Pittsburgh, PA 15213, USA; Tepper School of Business, Carnegie Mellon University, 5000 Forbes Avenue, Pittsburgh, PA 15213, USA

**Keywords:** human–AI teaming, complementarity, alignment

## Abstract

As artificial intelligence (AI) becomes embedded in critical decisions involving health, safety, finance, and governance, the key challenge is no longer whether humans and AI will collaborate, but rather how to structure this collaboration to achieve true complementarity. Human–AI complementarity refers to the conditions under which human–AI teams outperform either humans alone or AI systems alone. This paper advances the science of human–AI teaming for decision making by integrating insights from cognitive science, AI, human factors, organizational behavior, and ethics. We propose a framework grounded in collective intelligence and anchored in the foundational cognitive processes–reasoning, memory, and attention–to understand and engineer effective human–AI teams. We examine the sociotechnical factors that shape team effectiveness, including team composition, trust calibration, shared mental models, training, and task structure. We then outline design principles for achieving complementarity: defining goals and constraints, partitioning roles, orchestrating attention and interrogation, building knowledge infrastructures, and establishing continuous training and evaluation. We conclude with theoretical, practical, and policy implications, emphasizing alignment with human values, accountability, and equity. Together, these insights offer a roadmap for building human–AI teams that are not only high-performing and adaptive, but also transparent, trustworthy, and fundamentally human-centered.

## Introduction

Artificial intelligence (AI) is becoming deeply embedded in collective decision making that affects health, finance, safety, education, and governance (1). In these high-stakes domains, from medicine to public safety, deployments of AI often proceed without a clear theory of collaboration with humans, leading to issues such as erosion of human judgment (through automation bias or overreliance), diffusion of responsibility, and decisions that are faster yet harder to justify. To address these risks, researchers are increasingly making a distinction between AI as a team member rather than a mere tool (2–4). Human–AI teaming refers to 1 or more human agents and 1 or more AI agents working interdependently toward shared objectives (3).

We use the terms *team* or *teaming* to emphasize the process of collaboration between humans and AI, consistent with teamwork research that views teaming as an active, adaptive process, rather than a static structure (5). Viewing AI as a prospective teammate does not imply human-like agency or anthropomorphism, but it does highlight the need to clarify lines of authority and responsibility between humans and machines while finding synergies in their combined ability. The key challenge is how to design human–AI teaming in which humans and AI work together effectively and ethically to achieve complementarity, in which neither human teams nor AI teams could obtain better outcomes than humans and AI together (4, 6).

Recent advances in AI capabilities have created an inflection point in human–AI teaming. Whereas early AI systems were limited to automating routine tasks, today's AI can exhibit greater autonomy, adaptability, and even interactive conversational abilities (1). AI systems now occupy a spectrum of roles, from simple instruments at one end to dynamic partners at the other. Along this spectrum, AI's contribution to a team can vary in flexibility (from static to adaptive behavior) and agency (from reactive to autonomous decision making) (7). For example, some AI decision aids function as static tools that provide recommendations which humans may accept or ignore, whereas more adaptive teammates dynamically adjust their suggestions in response to human actions or changes in the environment (8, 9). In all cases, the goal of human–AI teaming is to complement each other (4, 6) otherwise, one could simply rely on humans alone or on AI alone, rather than using a team.

However, empirical evidence of human–AI complementarity is mixed and highly context dependent. Recent meta-analyses (6) suggest that hybrid human–AI teams can outperform either human teams or algorithmic systems alone, but only when collaboration is well calibrated and when humans understand when and how to rely on AI input. In other cases, poorly designed interaction or overreliance on algorithmic advice can lead to worse performance than either humans or AI working independently. Thus, although humans bring contextual understanding, intuition, and ethical reasoning, and AI contributes speed, scalability, and pattern recognition in large and high-dimensional datasets, achieving true complementarity remains an empirical challenge (4, 6). Recognizing when an AI functions merely as a tool and when it takes on the role of a teammate, and designing for that distinction, is essential for leveraging complementary strengths while maintaining human accountability (10). The key is not simply combining human and machine outputs, but also creating systems and workflows that support adaptive, transparent, and trust-calibrated human–AI teamwork (11, 12).

This paper advances the science of human–AI teaming for decision making by offering a unified theoretical framework grounded in recent work on collective intelligence (11). While prior work has proposed taxonomies of AI roles, trust factors, or hybrid intelligence patterns, we integrate insights from cognitive science, human factors, organizational behavior, AI alignment, and explainable AI within a collective intelligence framework organized around 3 foundational cognitive functions (reasoning, memory, and attention) and the meta-coordination processes that bind these components. This framework provides a common vocabulary for diagnosing strengths and limitations on both sides of the team, identifying when hybrid teams are likely to succeed or fail and specifying design principles for constructing effective human–AI teams.

Using this lens, we identify how AI systems can augment these functions within human–AI teams, helping them overcome common barriers to coordination, shared understanding, and informed decision making. Building on this framework, we clarify the conditions under which human–AI teams can achieve complementarity. We synthesize the sociotechnical factors that shape this complementarity, including team composition, trust calibration, shared mental models, training, and task structure. Finally, we translate these insights into design principles for constructing effective human–AI teams, including defining goals and constraints, partitioning roles, orchestrating attention and interrogation, building knowledge infrastructures, and establishing continuous training and evaluation. We conclude by outlining the theoretical, practical, and policy implications of this approach, emphasizing accountability, value alignment, and equity.

## Dimensions for human–AI team complementarity

Table 1 illustrates key dimensions of human–AI complementarity within a collective intelligence framework, organized around reasoning, memory, attention, as well as the meta-coordination and governance processes that structure and integrate these functions within a team. Each dimension underscores a common theme: by recognizing distinct strengths and limitations of AI systems and human teammates, and deliberately designing their complementarity, teams become greater than the sum of their parts. This framework may guide researchers and practitioners on where to focus when building or evaluating a human–AI team so that human intuition, creativity, and values synergize with machine precision, scalability, and consistency.

**Table 1.** Dimensions for human–AI complementarity highlighting the strengths and weaknesses of AI and humans and the opportunities to achieve complementarity.

Reasoning: AI acts as a structured facilitator, critic, and consistency engine: clarifying goals, surfacing assumptions, tracking trade-offs, and flagging misalignments. Humans provide contextual understanding, ethical judgment, accountability, and critical scrutiny.			
Dimension	AI strengths and weaknesses	Human strengths and weaknesses	Human–AI complementarity
1. Ethical authority and accountability	Strong at rule enforcement, logging, and consistency; **weakness:** lacks intrinsic values, cannot bear responsibility.	Moral intuition, value trade-offs, legitimacy to be held responsible; **weakness:** variable, influenced by bias and pressure.	Humans set goals/constraints and sign off on consequential decisions; AI provides auditable traces and checks policy compliance.
2. Explainability and transparency	Can provide structured rationales, confidence scores, and evidence links; **weakness:** opaque internals or misleading post hoc explanations.	Narrative sense-making and naturalistic explanation; **weakness:** illusion of explanatory depth, overconfidence.	Interfaces pair model evidence/confidence with human rationales; promote interrogation rather than blind acceptance.
3. Bias and fairness checks	Scales audits across large decision sets; **weakness:** inherits dataset and objective biases.	Empathy and norm awareness; **weakness:** implicit bias, inconsistency.	AI flags disparities and counterfactuals; humans define fairness criteria and adjudicate flagged cases.
4. Goal alignment and facilitation	Persistently tracks stated objectives and constraints; **weakness:** proxy drift.	Clarifies evolving goals and stakeholder values; **weakness:** hidden agendas, drift.	AI prompts goal clarification, surfaces conflicts; humans negotiate trade-offs and update objectives.
5. Error detection and recovery	Consistent checks and logging; **weakness:** overconfident failures.	Metacognition and reflective judgment; **weakness:** miss routine errors under fatigue.	Dual-layer defense: AI flags anomalies and inconsistencies; humans adjudicate, with explicit defer/override thresholds.

Memory: AI serves as a structured, context-aware memory engine. Humans preserve semantic meaning, judgment, and institutional legitimacy.			
Dimension	AI strengths and weaknesses	Human strengths and weaknesses	Human–AI complementarity
6. Knowledge storage and retrieval	Vast, rapid, verifiable retrieval with provenance; **weakness:** hallucinations, outdated sources.	Tacit, experiential, contextual knowledge; **weakness:** limited capacity and imperfect recall; turnover.	AI acts as a searchable institutional memory; humans validate, contextualize, and curate knowledge.
7. Expertise mapping/transactive memory	Learns who knows what and routes queries; **weakness:** cold-start issues, misclassification.	Awareness of skills and reputations; **weakness:** siloed visibility, biased perceptions.	AI maintains and updates expertise maps; humans correct, confirm, and adjust role assignments.

Attention: AI provides scanning, prioritization, misinformation filtering, and early-warning projections. Humans contribute broad contextual understanding, pattern novelty detection, and judgment about when and how to redirect collective attention.			
Dimension	AI strengths and weaknesses	Human strengths and weaknesses	Human–AI complementarity
8. Filtering, triage, and anomaly detection	Monitors streams at scale; **weakness:** false alarms, blind to “unknown unknowns,” alert fatigue.	Detects edge cases and interprets context; **weakness:** bounded bandwidth and fatigue.	AI handles routine vigilance; humans focus on exceptions and integrate broader situational cues.
9. Workload and focus orchestration	Coordinates timing, sequencing, and escalation; **weakness:** brittle to novel coupling or unforeseen dependencies.	Dynamic reprioritization and improvisation; **weakness:** coordination overhead.	AI proposes prioritization and handoffs; humans confirm, adapt, and re-plan.

Meta-coordination and governance (cross-cutting): AI executes structured processes and upholds procedural reliability through monitoring, workflow coordination, and auditability. Humans provide flexibility, creative restructuring, and norm-setting and design the overarching team architecture, including escalation paths, decision rights, and division of labor.			
Dimension	AI strengths and weaknesses	Human strengths and weaknesses	Human–AI complementarity
10. Team structuring and process	Scalable replication and reliable module execution; **weakness:** limited capacity for self-reorganization.	Adaptive role negotiation and creative restructuring; **weakness:** variability across teams and contexts.	Humans design team structures, adapt roles, and manage exceptions; AI ensures reliable execution, monitoring, and auditability.

In what follows, we highlight empirical findings and examples from multiple domains (healthcare, military, emergency response, corporate decision making, education) to point to the strengths and weaknesses in AI and human teammates, and to suggest ways of achieving human–AI complementarity.

### Reasoning

Reasoning in human–AI teams is fundamentally shaped by questions of ethical authority and accountability. These teams must determine not only how information is interpreted and decisions are reached, but also who has the legitimate authority to make value judgments, justify trade-offs, and bear responsibility (13). AI systems can provide consistency in rule enforcement and maintain detailed logs of decision pathways, but they cannot hold moral agency or be held responsible for harm (14, 15). Humans, by contrast, contribute moral intuition, contextual judgment, and the legitimacy required to adjudicate competing values, even though their reasoning is susceptible to biases and social pressures (16). This asymmetry makes complementarity essential: humans must remain the locus of ethical authority and accountability in consequential decisions, while AI can strengthen collective reasoning by clarifying goals, surfacing assumptions, flagging misalignments, and generating alternative hypotheses (17–20).

These ethical constraints also shape the degree of agency an AI teammate can appropriately have. In some contexts, such as safety-critical or value-laden decisions, AI systems must function as low-agency advisors whose role is limited to structuring information and highlighting inconsistencies, because human accountability cannot be delegated (14–16). In other contexts, AI tools may be granted higher levels of procedural agency (eg initiating alerts, coordinating information flow, or prompting reconsideration of options) when doing so enhances the team's reasoning without displacing human ethical oversight (17, 21). Emerging work on AI reasoning demonstrates how such a bounded agency can support team processes: large language models can be designed to detect goal conflicts, prompt clarification of priorities, summarize debates, generate alternative options, or act as a “devil's advocate” to surface hidden assumptions (22–24). Experimental systems show that these interventions help teams uncover overlooked possibilities and resolve inconsistencies in complex decisions. For example, AI monitors that ask targeted clarifying questions during democratic deliberation can help participants identify shared ground on contentious issues (25). These developments illustrate how AI can enhance human reasoning within ethically constrained forms of agency, supporting, rather than displacing, human authority and responsibility.

Explainability and transparency remain central to effective joint reasoning. Although modern AI models can provide structured rationales or confidence scores, their internal processes often remain opaque, and post hoc explanations can be incomplete or misleading (26). On the other hand, humans naturally craft narrative explanations but frequently overestimate the depth of their understanding (27). Empirical research shows that teams perform best when humans actively interrogate AI recommendations rather than passively accepting them (6). In clinical settings, for instance, teams outperform either humans or AI alone when clinicians are trained to question AI outputs and the system supplies evidence-based justifications (28). AI can further support explainability by prompting users to articulate their reasoning, juxtaposing human and machine rationales, and identifying inconsistencies or missing premises (25, 29).

Bias and fairness concerns also rely heavily on shared reasoning. AI systems can identify disparities, counterfactual inconsistencies, or subtle patterns of discrimination across large datasets (30, 31). At the same time, AI models can inherit or reproduce structural biases from their training data, necessitating vigilant human oversight (32, 33). Humans bring empathy and contextual norms to fairness judgments but remain vulnerable to implicit biases and inconsistent evaluation (34). Facilitative AI agents can support fairness by simulating alternative viewpoints, highlighting underrepresented perspectives, or signaling when deliberation drifts toward narrow or biased frames (35). Ultimately, fairness decisions still require human adjudication, but AI can significantly enhance the detection and early surfacing of fairness-relevant concerns.

Goal alignment under uncertainty offers another domain where human and AI reasoning complement one another. Research on AI alignment demonstrates how proxies and objectives can drift as contexts change, leading to misaligned system behavior (36–38). Humans are skilled at reconciling shifting stakeholder values but often struggle with value drift or inconsistent prioritization (12). AI systems can assist by tracking evolving constraints, focusing attention on high-impact decisions, and signaling inconsistencies between stated goals and observed actions (39). AI facilitators can also manage group discussions by highlighting trade-offs, summarizing areas of disagreement, and identifying potential compromise solutions (23, 24). Effective design additionally includes explicit value modeling and conflict resolution protocols to keep AI behavior aligned with team goals while ensuring humans remain engaged as critical evaluators (10, 40).

Finally, error detection and recovery illustrate the dual strengths of humans and AI in reasoning processes. AI systems excel at systematic checks, anomaly detection, and consistency monitoring but may fail silently with high confidence when encountering unfamiliar conditions (41). Humans provide metacognitive oversight and contextual reasoning, though their vigilance degrades under fatigue and cognitive load (42). Together, layered defenses supported by interfaces that foreground uncertainty, highlight anomalous interaction patterns, or prompt reconsideration create a more reliable joint reasoning system (43). AI agents can detect emerging anomalies in team communication or coordination, alerting humans to potential breakdowns before they escalate (39), while humans adjudicate the significance and appropriate response.

In sum, complementarity in reasoning emerges when AI acts as a structured facilitator, critic, and consistency engine: clarifying goals, surfacing assumptions, tracking trade-offs, and flagging misalignments, while humans provide contextual understanding, ethical judgment, accountability, and critical scrutiny. Designing AI to support these joint processes, while preserving human interrogation, rather than deference, allows human–AI teams to achieve more resilient and higher-quality reasoning than either alone (25).

### Memory

Memory in human–AI teams encompasses how knowledge is stored, retrieved, and distributed across individuals, technologies, and the collective. In human groups, this function is grounded in collective memory, the aggregation and coordination of knowledge held across team members and managed through transactive memory systems, the shared understanding of who knows what and who is responsible for specific information (44–47). Well-developed transactive memory enables teams to more quickly access diverse expertise and perform better, yet many human teams underutilize available knowledge, overlook teammates’ expertise, or overrely on external searches that create overload (48, 49). Classic work demonstrates that teams with coordinated knowledge structures consistently outperform those with unaligned or redundant memory systems (44, 50), but redundancy, gaps in expertise awareness, and underuse of institutional knowledge remain common pitfalls.

AI systems offer the potential to strengthen and extend collective memory by serving as knowledge amplifiers, curators, and organizers. Their high-capacity storage and rapid retrieval allow them to act as institutional memory repositories that can recall past decisions, lessons learned, and relevant precedents, reducing the likelihood of repeated mistakes (51). AI-augmented memory systems can map and track expertise, making collaborators aware of “who knows what” even in distributed or virtual teams in which visibility is low (52, 53). By indexing role-relevant information, detecting expertise gaps, and proactively surfacing contextually appropriate knowledge, these systems can reduce time to insight and support more coordinated decision making (54, 55). Moreover, AI memory functions reinforce and support collective reasoning by retrieving information aligned with evolving goals or flagging when new evidence suggests a shift in priorities.

However, AI's prodigious memory is only as useful as its accuracy, sourcing, and currency. Language models may hallucinate or present outdated information with confidence, making human oversight essential to verify and contextualize machine-provided knowledge (56, 57). Humans contribute tacit, experiential, and embodied knowledge, which are forms of know-how that AI does not possess and that are crucial for interpreting ambiguous or novel situations (58). When combined with human judgment, “cognitive prosthetics” such as AI-enhanced checklists and decision aids can reduce errors and improve recall of critical information (59). These hybrid approaches illustrate the value of coupling high-capacity machine memory with human interpretive intelligence.

Hybrid knowledge systems in which AI retrieves, organizes, and cross-references information while humans validate, contextualize, and adjudicate are emerging as a robust model for organizational learning and knowledge governance (60, 61). In such systems, AI provides provenance, confidence estimates, and expertise maps, while humans act as custodians who vet sources, interpret edge cases, and integrate tacit experience. Complementarity emerges when AI serves as a structured, context-aware memory engine and humans preserve semantic meaning, judgment, and institutional legitimacy. Designing AI systems to expose provenance, track expertise, and maintain shared repositories updated by both humans and machines can strengthen transactive memory over time and contribute to more resilient, high-performing human–AI collectives.

### Attention

Attention concerns how human–AI teams monitor information, handle exceptions, and allocate focus in dynamic environments. At the collective level, collective attention depends on the amount of undivided focus team members give to shared work and the degree to which that focus is aligned with group priorities (47). Within the team action process framework, attention encompasses monitoring progress, detecting errors, revising priorities, and adapting as conditions change (62). These processes have become increasingly challenging in today's data-rich, misinformation-prone environments, in which failures of collective attention such as overlooked indicators or miscommunication during changing conditions are a leading cause of errors and accidents, especially given human susceptibility to fatigue, distraction, and overload (63–65).

AI tools can augment team monitoring capacity by filtering and triaging large data streams, flagging misinformation, detecting anomalies in real time, and coordinating focus among team members with speed and consistency (66, 67). These systems can also anticipate diverse interests through stakeholder perspective taking (20), run scenario-based diagnostics to uncover blind spots (68), and track workload and progress to prompt adjustments when tasks become unbalanced (11, 13). Functionally, this creates something akin to a transactive attention system in which AI dynamically allocates collective attention as priorities shift, elevating the most urgent or high-impact information.

Research in human–autonomy teaming demonstrates that well-designed AI alerts can significantly improve hazard detection rates, though poor alert design can create new vulnerabilities such as complacency or alert fatigue (69). Clear role definitions and closed-loop communication protocols help integrate AI-generated signals into team workflows so that critical information is acknowledged, acted upon, and verified (70, 71). When AI performs routine vigilance and humans focus on ambiguous, context-sensitive, or novel edge cases, the hybrid system achieves more robust monitoring and interpretation, an insight consistent with work in hybrid intelligence (72).

Yet the goal is not to replace human attention but to structure complementarity. Overreliance on AI may cause humans to disengage from active monitoring, increasing the likelihood that unexpected or unusual cases go unnoticed. AI provides unfaltering consistency but often struggles with “unknown unknowns,” while humans are prone to miss routine signals but excel at detecting anomalies or weak signals that require contextual interpretation (65, 72, 73). Balanced, transparent alerting that indicates why an item deserves attention helps sustain human vigilance and enables better assessment of alert validity.

Workload orchestration is another central attentional function. AI can propose prioritizations, sequence tasks, allocate work across the team, and manage escalation pathways, though its coordination strategies can become brittle in unforeseen or rapidly evolving situations (74). Humans excel at improvisation, dynamic replanning, and navigating unstructured environments, albeit with higher coordination costs. Empirical work on human–AI teaming shows that AI-generated prioritization combined with human oversight leads to more adaptive coordination and higher collective performance (75).

To achieve complementarity, AI should provide always-on scanning, prioritization, misinformation filtering, and early-warning projections that help teams rise above noise and maintain situational awareness. Humans, meanwhile, contribute broad contextual understanding, pattern novelty detection, and judgment about when and how to redirect collective attention. Structuring roles, acknowledgement protocols, and adaptive alerting mechanisms can fuse machine vigilance with human perceptiveness into sustained collective attention and resilient team performance (62).

### Meta-coordination and governance

Meta-coordination and governance processes shape how reasoning, memory, and attention are organized and integrated. Research on collective intelligence emphasizes that team structures, interaction protocols, and role clarity strongly influence group performance (76, 77). AI systems excel at executing structured processes consistently and at scale but are limited in their ability to reorganize themselves or adapt internal roles. Humans provide flexibility, creative restructuring, and norm setting, though they vary widely in reliability and may struggle to maintain coherence in large or distributed teams (78).

Hybrid governance emerges when humans design the overarching team architecture, including escalation paths, decision rights, and division of labor, while AI upholds procedural reliability through monitoring, workflow coordination, and auditability (75). This creates a governance structure in which human judgment guides system-level adaptation and AI ensures operational consistency. Meta-coordination therefore functions as the connective tissue that binds reasoning, memory, and attention into an integrated, high-performing human–AI collective.

## Factors shaping human–AI complementarity in hybrid teams

Having outlined the cognitive and structural foundations of human–AI teaming, we now turn to the factors that shape when and how these foundations support complementarity in practice. The effectiveness of human–AI teams depends heavily on internal factors: team composition, coordination dynamics, individual characteristics, and interaction patterns that must dynamically respond to external demands. These factors determine when human–AI complementarity is likely to succeed.

### Team composition and size

Team size and composition are critical, as larger teams can increase coordination complexity (79). Recent work has shown that human–AI teams with humans in the minority can experience reduced trust and weaker shared knowledge (80–82), both associated with diminished performance (83, 84), especially in highly interdependent tasks (85). Role clarity and specialization mitigate these risks: teams perform better when each member (human or AI) has defined responsibilities that leverage their strengths (86). In practice, this may mean pairing humans with AIs in complementary roles (eg human strategy with AI analysis) while ensuring that humans understand what each AI does and why.

### Trust calibration and shared mental models

Effective teaming with AI requires calibrated trust and accurate shared mental models of the AI's abilities, limitations, communication style, and monitoring needs (65, 87). Incomplete or divergent mental models lead to delegation errors and coordination breakdowns (88–91). Calibrated trust avoids both undertrust (algorithm aversion) and overtrust (complacency). Humans are less likely to rely on AI when the stakes are high, due to perceived risk (92), and trust can drop sharply after witnessing an AI error (93). Ambiguity about accountability can exacerbate reluctance to delegate, though salient AI errors can sometimes aid learning and improve calibration over time (94).

Well-established human factors results apply: trust depends on the AI's reliability, transparency, and the user's prior experience and training (95), and trust correlates positively with team performance; interventions like communication and transparency help (85). Interfaces that expose uncertainty and rationales can strengthen shared mental models and improve calibration, communicating confidence and reasons for low confidence so that humans know when to scrutinize or defer (96).

### User expertise and workload

Effectiveness varies across individuals with task/AI expertise (97, 98), mental models of AI (91, 99, 100), and prior experiences with automation. Positive prior experiences predispose users to accept and integrate new AI systems; negative experiences do the opposite. Novices and experts may benefit from different collaboration modes (eg more guidance and guardrails for novices; “sparring partner” use for experts) (101). Other factors, such as fatigue, workload, time of day, and emotional state, can materially influence performance and reliance patterns (102). Preferences and values also matter; for instance, clinicians differ in tolerance for false positives versus false negatives, which AI tools must accommodate (34).

### Task characteristics

Not all tasks are equally amenable to human–AI teaming. Evidence suggests the largest gains occur in complex, uncertain tasks where human and AI error patterns differ and can offset each other (6). In well-defined, highly structured tasks, AI alone often excels; in open-ended, strategic tasks rich in tacit context, humans retain an advantage. The “sweet spot” includes classification, prediction, and diagnosis under uncertainty (eg medical diagnosis in which AI contributes pattern recognition and humans integrate patient context and values). Contextual factors shape reliance: humans are less likely to accept AI recommendations for high-stakes decisions, in part due to accountability concerns (103). Time pressure and fatigue also shift dynamics: under pressure, people may lean on AI for speed—or bypass it if it slows them—while fatigue can both increase human errors (raising AI's relative value) and reduce responsiveness to AI alerts (104).

## Constructing complementary human–AI teams

The design principles that follow map directly onto the collective intelligence framework outlined previously: defining goals supports reasoning, knowledge infrastructures support memory, attention orchestration supports collective attention, and role partitioning and training underpin meta-coordination.

### Define goals and constraints

Defining goals and constraints strengthens collective reasoning by ensuring that human values, trade-offs, and ethical boundaries are explicit. Designers should align team actions with human-defined objectives and ethical principles from the outset by encoding multiobjective goals and guardrails (eg fairness, accountability, safety) ensuring that AI systems optimize task performance while remaining constrained by these value-protecting requirements (105). International frameworks call for transparency, fairness, and accountability; in teams, this implies audit trails, interpretable reasoning, circuit breakers requiring human review for ethically sensitive actions, and clear mechanisms for recourse. During rapidly changing contexts (eg pandemics), tools must update to new realities while humans adjust reliance based on current performance (40, 98). Clear expectation setting mitigates misaligned objectives and overreliance (91 ). Reliability and explainability remain essential to trust, especially in high-stakes domains (106).

### Define knowledge infrastructure

Knowledge infrastructures reinforce collective memory through five core design elements: (i) seamless workflow integration that enhances rather than disrupts existent work practices; (ii) transparent and auditable data and model pipelines, including provenance tracking, interpretability, and fairness audits (33); (iii) mechanisms for continuous updating in dynamic environments (40, 98) and cross-domain learning (eg insights from autonomous vehicles informing agricultural robotics); (iv) collaborative artifacts such as shared dashboards, audit trails, and traceable decision records, that enable auditing, adjustment, and calibrated reliance; and (v) intuitive interfaces and communication channels (eg conversational queries, context-sensitive presentation) that minimize cognitive load and prevent mode confusion while keeping humans in control. Reliability and graceful failure modes (eg fallback to human-only operation, smooth handoffs) further preserve safety and trust under uncertainty (107).

### Implement attention and interrogation orchestration

Attention and interrogation orchestration operationalize a shared attention system by specifying how information is triaged, when AI outputs should be questioned, and how escalation decisions is triggered. Interaction should be designed so that AI elevates signal over noise, while humans interrogate outputs and guide higher-level reasoning. AI can triage data streams, flag anomalies, and maintain situational awareness under time pressure, whereas humans question assumptions, weigh competing values, and decide when to override. Interfaces should make uncertainty and underlying rationales explicit to support active interrogation rather than passive acceptance (99, 108). Teams should establish escalation and disagreement protocols (eg confidence thresholds for deferring to humans; concurrence requirements for critical actions) and treat errors as learning opportunities through after-action reviews that incorporate both human and AI system outputs (109). This orchestration reduces over- or under- trust (algorithm aversion) and curbs automation complacency (93, 110) while leveraging the benefits of conspicuous errors for calibration (94). Process-aware measurement (eg goal alignment, division of labor) helps identify bottlenecks and refine orchestration (6, 111).

### Partition roles

Partitioning roles establishes clear decision rights and interaction protocols across humans and AI, strengthening meta-coordination and governance. Complementarity request deliberately assigning responsibilities so that each party performs the functions that it is best suited for (112). For example, in loan approvals, AI systems can conduct large-scale data analysis and risk scoring, while human officers adjudicate borderline cases and communicate decisions, preserving empathy, contextual judgment, and accountability. At critical junctures, humans should retain decision authority (human-in-command/on-the-loop), with AI supporting pre/postprocessing analysis and monitoring execution. Clear role allocation enhances transparency and compliance by making it traceable which components were generated by AI versus humans, while also reducing coordination friction. This approach aligns with human-centered AI principles that emphasize augmentation rather than replacement (113).

### Training and evaluation

Training and evaluation enables human–AI teams to refine shared mental models, calibrate trust, and adjust division of labor over time. Together, these principles translate the cognitive foundations of human–AI teaming into concrete practices for achieving complementarity in real-world settings. Humans need practice interpreting AI outputs, knowing when to override, and coordinating under time pressure (99, 108). AI systems should adapt to human feedback (eg chatbots learning from corrections, reinforcement learning from human feedback). Shared training environments and simulations (eg air traffic management, emergency response) build fluency and trust. Iterative team-lab prototyping has shown success in domains such as mixed-initiative planning for space missions (114). Teams should evaluate not only outcomes (speed, accuracy, trust), but also process quality (goal alignment, division of labor, workload balance, error recovery rate) to detect fragilities that aggregate performance metrics may obscure (6, 111). After-action reviews should be used to update both AI models and human protocols, adapting escalation thresholds and interface behaviors as teams mature. Over time, these feedback cycles improve robustness, fairness, and user acceptance, yielding human–AI teams that are high-performing, adaptable, trustworthy, and aligned with human values.

## Conclusions and recommendations

The rise of human–AI teaming marks a profound transformation in how decisions are made across domains, from healthcare and emergency response to finance, transportation, and governance. These hybrid systems hold the promise of truly complementary decision making, in which the speed, consistency, and scalability of AI are harmonized with the contextual awareness, ethical reasoning, and adaptability of human cognition. Realizing this potential, however, requires deliberate design, rigorous evaluation, and principled governance.

Human–AI teams must be purposefully structured for complementarity, grounded in a deep understanding of the domain, the nature of the task, and the distinct strengths and limitations of each partner. AI systems should augment, rather than replace, human judgment, assuming roles aligned with their computational advantages while deferring to human oversight in areas demanding ethical discretion, contextual sensitivity, or accountability (4, 115–118). Seamless integration into existing workflows, supported by transparent and interpretable interfaces, ensures that these systems enhance, rather than disrupt, human performance.

Theoretically, advances in human–AI teaming benefit from grounding in team science and collective intelligence constructs such as transactive memory, attention, and reasoning, which provide a framework to understand how humans and AI share knowledge, coordinate focus, and co-reason toward common goals. Future research should extend this theoretical foundation through longitudinal studies, real-world evaluations, and benchmarks assessing not only accuracy, but also trust calibration, shared understanding, and ethical alignment. Interdisciplinary collaboration among AI developers, cognitive scientists, human factors experts, and ethicists will be essential to bridge the remaining gaps in understanding, particularly around alignment of AI behavior with nuanced human values and the design of AI as active, adaptive teammates (119, 120).

Practically, organizations must invest in joint training that builds trust, interpretability, and fluency between humans and AI before deployment. Humans need not only technical literacy, but also cognitive readiness to interpret and, when necessary, challenge AI outputs, while AI systems must learn from human feedback to refine their models (99, 108). Metrics of team success should move beyond performance accuracy to include indicators of collaboration quality, workload balance, and resilience (6, 111). In dynamic decision environments, flexible evaluation frameworks are crucial as fixed benchmarks can quickly become obsolete (4).

Although our framework synthesizes empirical findings across multiple fields, much of the existing evidence for human–AI complementarity comes from laboratory studies, small-scale deployments, or early-stage prototypes. The generalizability of these results to high-stakes, long-term, or highly heterogeneous environments remains uncertain. Future research will require longitudinal field studies, shared benchmarks that evaluate collaboration processes rather than accuracy alone, and deeper investigation into how human–AI teams evolve, adapt, and maintain alignment over time (4).

Policymakers and regulators have a central role in ensuring that the deployment of human–AI teams aligns with societal values. Governance frameworks should enforce transparency, fairness, and accountability without stifling innovation. Clear standards for explainability, auditability, and human oversight can safeguard the public interest, particularly in high-stakes domains such as healthcare or criminal justice (33, 106). Public institutions must proactively communicate how AI is involved in decisions that affect individuals, ensuring inclusivity and trust through openness and recourse mechanisms. Sustained policy attention should support ongoing research, cross-sector learning, data and measurement infrastructure for designing and evaluating human–AI teaming, and incident reporting systems that encourage collective improvement across industries.

In sum, the creation of effective human–AI teams represents not just a technical challenge, but also a societal transformation. Achieving their promise will require aligning design and training practices with ethical and institutional safeguards, fostering public trust through transparency, and advancing a science of human–AI teaming that balances innovation with accountability. If pursued thoughtfully, human–AI collaboration can yield decisions that are not only faster and more scalable, but also more just, adaptive, and profoundly human-centered.

## Data Availability

There are no data underlying this work.
